# Mycorrhizal inoculation enhanced tillering in field grown wheat, nutritional enrichment and soil properties

**DOI:** 10.7717/peerj.15686

**Published:** 2023-09-13

**Authors:** Muhammad Akbar, Safeer A. Chohan, Nasim A. Yasin, Aqeel Ahmad, Waheed Akram, Abdul Nazir

**Affiliations:** 1 Department of Botany, University of Gujrat, Gujrat, Punjab, Pakistan; 2 SSG, RO-II Department, University of the Punjab, Lahore, Punjab, Pakistan; 3 University of Chinese Academy of Sciences, Beijing, Beijing, China; 4 Department of Plant Pathology, University of the Punjab, Lahore, Punjab, Pakistan; 5 Department of Environmental Sciences, COMSATS University Islamabad, Abbottabad, Pakistan

**Keywords:** Mycorrhizae, Wheat, *Claroideoglomus lamellosum*, Funneliformis mosseae

## Abstract

To meet food security, commercial fertilizers are available to boost wheat yield, but there are serious ill effects associated with these fertilizers. Amongst various organic alternatives, inoculating crop fields with mycorrhizal species is the most promising option. Although, mycorrhizae are known to enhance wheat yield, but how the mycorrhizae influence different yield and quality parameters of wheat, is not clear. Therefore, this study was undertaken to investigate the influence of indigenous mycorrhizal species on the growth of wheat, its nutritional status and soil properties, in repeated set of field experiments. In total 11 species of mycorrhizae were isolated from the experimental sites with *Claroideoglomus,* being the most dominant one. Five different treatments were employed during the present study, keeping plot size for each replicate as 6 × 2 m. Introduction of consortia of mycorrhizae displayed a significant increase in number of tillers/plant (49.5%), dry biomass (17.4%), grain yield (21.2%) and hay weight (16.7%). However, there was non-significant effect of mycorrhizal inoculation on 1,000 grains weight. Moreover, protein contents were increased to 24.2%. Zinc, iron, phosphorus and potassium concentrations were also increased to 24%, 21%, 30.9% and 14.8%, respectively, in wheat grains. Enhancement effects were also noted on soil fertility such as soil organic carbon % age, available phosphorus and potassium were increased up to 64.7%, 35.8% and 23.9%, respectively. Herein, we concluded that mycorrhizal introduction in wheat fields significantly increased tillering in wheat and this increased tillering resulted in overall increase in wheat biomass/yield. Mycorrhizae also enhanced nutritional attributes of wheat grains as well as soil fertility. The use of mycorrhizae will help to reduce our dependance on synthetic fertilizers in sustainable agriculture.

## Introduction

Wheat is the staple food of populace in many countries. Annual production of wheat in Pakistan was 25.2 million tons, during years 2019–20. Extensive use of synthetic fertilizers *e.g.*, urea, phosphate and potash (NPK) to enhance the yield of wheat has beneficial effects on one hand considering yield of wheat but on the other side, it also poses number of ill effects Like environmental pollution and human health concerns (Lopes-Ferreira et al., 2022). In recent years, there is increasing demand for nature friendly fertilizers *e.g.*, mycorrhizal formulations (Messa & Savioli, 2021; Delvian & Hartanto, 2021; Nasiyev et al., 2021). Mycorrhiza plays a key role in nutrient cycling in ecosystem, and protects host plant against environmental stress (Zhang et al., 2003; Huey et al., 2020).

Mycorrhizal biotechnology has become a major component of sustainable agriculture (Srivastava, Johny & Adholeya, 2021; Schultz, Wu & Baumann, 2022). Plant symbiosis with arbuscular mycorrhizal fungi (AMF) yields numerous benefits to host plant, including enhanced nutrient uptake (Stahlhut et al., 2021). The majority of land plants form symbiotic mycorrhizal associations with AMF and a single plant species may be colonized by a wide range of phylogenetically diverse AMF species (Säle et al., 2021). The major advantage of mycorrhizae to crops is improved soil nutrients and water uptake (Noceto et al., 2021). AMF are well documented to have enhancement effects on crop growth, especially in phosphorus deficient soils. Mycorrhizal fungi increase soil phosphorus (P), nitrogen (N) and soil organic carbon (Kilpeläinen et al., 2016; Nahar, Bovill & McDonald, 2021). These mycorrhizal fungi also aid in N recovery from the nitrogen fertilizers (Ingraffia et al., 2020; Ingraffia et al., 2021). The application of multiple moderate doses of phosphorus and nitrogen acts as a stimulant for the maximum development of mycorrhizae in wheat plants (Vidican et al., 2020). Thus, the use of mycorrhizae can be helpful in reducing the use of synthetic fertilizers in the crops and this can help to overcome the hazardous effects of these synthetic chemical fertilizers.

In an investigation, the application of *Glomus fasciculatum* resulted in 22% significantly higher grain yield in wheat, but the increase in tillers/plant was less than control (Khan & Zaidi, 2007). The 1,000 grains weight was not reported in that study, so there was no conclusive evidence about the growth parameter that actually contributed to grain yield or total wheat biomass. Another report showed the effects of mycorrhizae on growth of wheat, in which mycorrhizae increased wheat yield by increasing various yield parameters in wheat *e.g.*, tillers/plant and 1,000 grains weight. Although they reported increase in tillering as well as 1,000 grains weight of wheat, but that study could not provide conclusive evidence because there was no treatment to compare the effects of mycorrhizal species when inoculated in the presence of urea (Seyedlar et al., 2014). Although, mycorrhizal fungi have shown promising results in terms of increase in biomass of wheat crop, but many studies regarding effects of mycorrhizal inoculation were carried out under pot conditions. Moreover, the exact growth parameter contributing to overall growth and grain yield in field grown wheat is not clear. Therefore, this study was undertaken to investigate the influence of indigenous mycorrhizal species on different growth parameters of wheat and their contribution to overall increase in grain yield, nutritional status of wheat as well as soil properties, in repeated set of field experiments.

## Materials & Methods

### Mycorrhizal species of study areas

Mycorrhizal species were identified from two locations. One location was a farmer’s (Muhammad Afzal) field at village Naka Kahut, Chakwal, Pakistan (32.9423°N latitude, 72.4787°E longitude, elevation 460 m), site-1 and the second location was a farmer’s (Muhammad Arif) field at village Malakwal, Chakwal, Pakistan (32.9132°N latitude, 72.3992°E longitude, elevation 374 m), site-2. Earthen pots were filled with soil collected from these study areas. Onion bulbs were grown in these pots and plants were watered as per requirement. After three weeks, onion plants were harvested and mycorrhizal species were identified with the help of spores. Spore isolation was done using the wet-sieving and decanting method (Gerdemann & Nicolson, 1963). The morphological identification of arbuscular mycorrhizal fungal spores was accomplished by adopting method described by (Schenck & Perez, 1990).

### Mass culturing of mycorrhizal strains

Spores of identified mycorrhizal species were collected. Then soil taken from experimental areas was sterilized in an autoclave (Hirayama, HVE-50, Tokyo, Japan) at 121 °C for 30 min and filled into earthen pots @ 6 kg pot^−1^. Then onion bulbs were grown in these pots, containing identified spores of mycorrhizae. Plants were watered as per plant requirements based on visual observations of potted plants and pot soil. After three weeks, onion plants were harvested. Fine roots of onion having mycorrhizal infections were collected and these roots were used @ 16.57 kg ha^−1^, (≈ 22 mycorrhizal propagules gram^−1^ of soil) in required treatment plots. Mycorrhizal dead inoculum was prepared by autoclaving the live mycorrhizal inoculum at 121 °C for 30 min. Mycorrhizal dead inoculum treatments were designed to investigate the effects of mycorrhizal biomass on the growth parameters of wheat.

### Selection of fertilizers and wheat variety

Inorganic fertilizers, NPK (8:23:18) and urea were purchased from the local market. Recommended doses of N (120 kg ha^−1^), P (26.2 kg P ha^−1^) and K (33.2 kg ha^−1^) for wheat were applied to all the plots according to treatments. N fertilizer was applied in two split doses *i.e.,* half at seedbed preparation and half at the flowering stage. Wheat variety, Faisalabad-2008, as recommended by Agricultural Department, Punjab, Pakistan was selected as test crop to investigate the effects of mycorrhizae on its growth.

### Soil analysis

Soil samples were taken at pre-sowing (before addition of fertilizers and mycorrhizal inocula) and post-harvest stages. Soil sampling was done for every replicate of every treatment plot of experimental areas at the depth of 15 cm and mixed well to get composite soil samples of each replica of each treatment. Analyses of the soil samples were done by adopting standard soil tests for following characteristics, *viz.*, soil texture, saturation percentage, soil pH, electrical conductivity of the saturation extract (ECdsm^−1^), soil organic carbon (SOC) % age, available phosphorus (P mg kg^−1^) and available potassium (K mg kg^−1^). The pH was measured with a pH meter, while electrical conductivity (EC) was measured on a conductivity meter. The phosphorus was measured by following Olsen & Sommers (1982). The SOC content was calculated by adopting standard procedure for SOC (Bao, 2000).

### Field preparation

Field trials (field study approval No. UOG/BOT/1) were conducted to investigate the effects of mycorrhizae under reduced dose of NPK. The fields were prepared using moldboard plow, disk and leveler. The furrows were formed by furrower to separate a plot from its neighboring ones, keeping plot size as 6 × 2 m. Experiments were repeated at two different sites as mentioned above, following the randomized complete block design (RCBD). Five treatments were investigated in the present study as followings: Control, where there was no addition of fertilizers or mycorrhizal inocula; Wheat + NPK full dose (FD); Wheat + NPK half dose (HD); Wheat + mycorrhizae alive + NPK half dose (MA+HD); Wheat + mycorrhizae dead + NPK half dose (MD+HD). Proper sowing method including time of sowing, depth of sowing (05 cm) and kernel rate @ 150 kg ha^−1^ and 22 cm row to row spacing were adopted. In all treatments, weeds were controlled by recommended commercial herbicides, fenoxaprop-P-ethyl and bromoxynil + 2-methyl-4-chlorophenoxyacetic acid (MCPA).

### Harvesting and data collection

Data regarding plant height and number of tillers/plant were recorded before harvesting. Harvesting was managed manually at maturity, after 175 days of sowing. The harvested wheat was sun-dried for one week, weighed with digital weighing machine and threshed with a thresher. Following parameters were recorded after harvest *viz.*, dry mass, grain yield, hay weight and 1,000 grains weight. The chemical composition of wheat grains was calculated following (AOAC, 1970). Concentrations of mineral elements were calculated by atomic absorption spectrophotometer. At harvest, composite soil samples were taken again from every replicate/treatment.

### Statistical analyses

All the data were analyzed by ANOVA & Tukey’s Test by using Minitab-19 software. More information about statistical analyses is given within description of each table/figure.

## Results

### Diversity of mycorrhizal species in study areas

**Figure 1:**
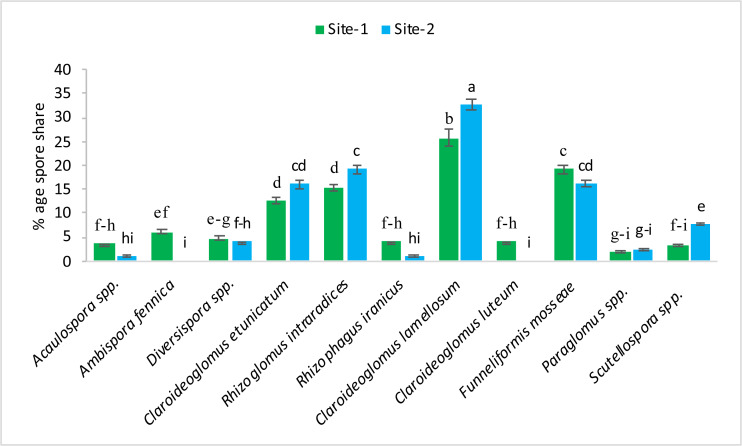
Pre-sowing percentage occurrence of mycorrhizal species at site-1 and site-2. Vertical bars represent standard error of means of three replications. Bars sharing same letter do not differ at *P* ≤ 0.05 as computed by ANOVA & Tukey’s test, using Minitab-19.

**Figure 2:**
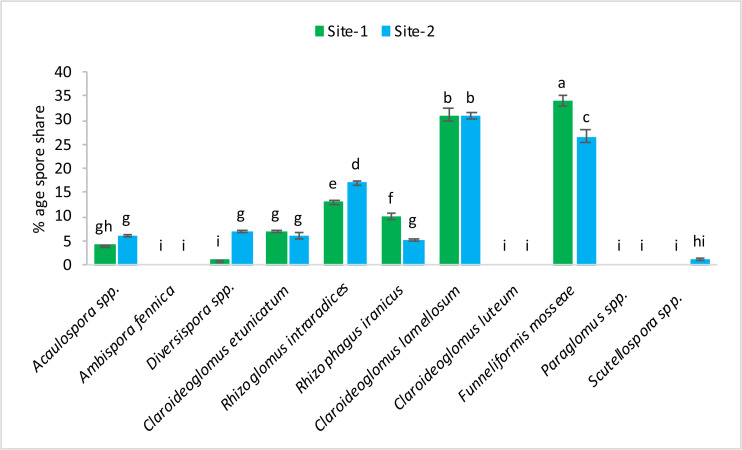
After harvest percentage occurrence of mycorrhizal species at site-1 and site-2. Vertical bars represent standard error of means of three replications. Bars sharing same letter do not differ at *P* ≤ 0.05 as computed by ANOVA & Tukey’s test, using Minitab-19.

The following 11 species of mycorrhizae were identified from study areas: *Acaulospora* spp., *Ambispora fennica, Diversispora* spp., *Claroideoglomus etunicatum, Rhizoglomus intraradices, Rhizophagus iranicus, Claroideoglomus lamellosum, Claroideoglomus luteum, Funneliformis mosseae, Paraglomus* spp., and *Scutellospora* spp. The mycorrhizal species, *Claroideoglomus lamellosum* and *Funneliformis mosseae* were found to be the dominant species in both experimental sites. The % age spore share of each species is presented in Figs. 1–2.

### Effects of mycorrhizae inoculation on height of wheat plants

**Figure 3:**
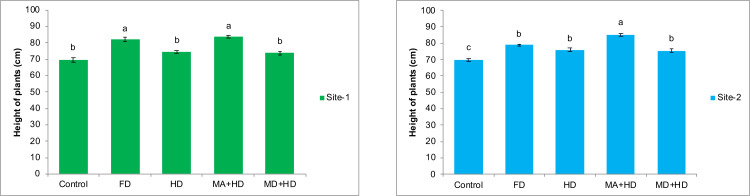
Effect of mycorrhizae on height of wheat plants at site-1 and site-2. Vertical bars represent standard error of means of three replications. Bars sharing same letter do not differ at *P* ≤ 0.05 as computed by ANOVA & Tukey’s test, using Minitab-19. FD. Wheat with full/recommended dose of NPK; HD. Wheat with half of recommended dose of NPK; MA+HD. Wheat with HD of NPK and mycorrhizal alive inoculum; MD+HD. Wheat with HD of NPK and mycorrhizal dead inoculum.

The results of combined effects of mycorrhizal inoculation with reduced doses of NPK fertilizers on height of wheat plants over non inoculated wheat (Control), recommended/full dose of NPK (FD), and half of recommended dose of NPK (HD), were found significant, at both experimental locations. At site-1, amongst all treatments, maximum significant increase in height of wheat plants was observed in mycorrhizae alive + half dose of NPK (MA+HD). There was 13.8% increase in plant height as compared to plots where half dose of fertilizer was used with dead mycorrhizal inoculum (MD+HD). MA+HD showed increase in height of plants by 12.22%, 2.4% and 20.6% over, HD, FD and control respectively. At site-2, MA+HD showed increase in height of wheat plants by 13%, 12.3% and 8% over MD+HD, HD and FD, respectively (Fig. 3).

### Effects of mycorrhizae inoculation on number of tillers/plant

**Figure 4:**
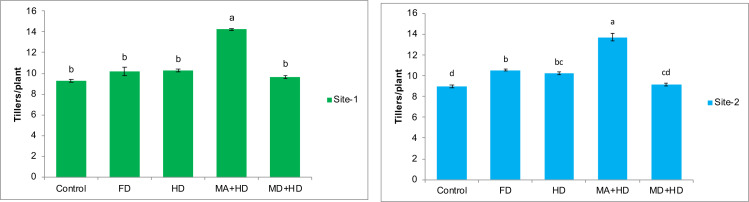
Effect of mycorrhizae on number of tillers/plant of wheat plants at site-1 and site-2. Vertical bars represent standard error of means of three replications. Bars sharing same letter do not differ at *P* ≤ 0.05 as computed by ANOVA & Tukey’s test, using Minitab-19. FD. Wheat with full/recommended dose of NPK; HD. Wheat with half of recommended dose of NPK; MA+HD. Wheat with HD of NPK and mycorrhizal alive inoculum; MD+HD. Wheat with HD of NPK and mycorrhizal dead inoculum.

The effects of different treatments on number of tillers/plant of wheat were found to be the most promising effects recorded in the present study. At site-1, there was 10%, 10.8%, 53.6%, and 3.96% increase in tillers/plant of wheat in treatments *viz.* FD, HD, MA+HD and MD+HD, respectively, as compared to plots where no NPK fertilizer as well as no mycorrhizal inoculations was made (Control). Amongst all treatments, significant increase in tillers was observed in MA+HD. There was 47.75% increase in tillers/plant as compared to MD+HD. MA+HD also showed an increase of 38.6% and 39.5% in number of tillers of wheat over, HD and FD, respectively (*r*^2^ = 96.21%, *F* = 65.52). At site-2, there was 17.5%, 14.5%, 52.8%, and 2.2% increase in tillers/plant of wheat in treatments *viz.* FD, HD, MA+HD and MD+HD, respectively, as compared to control plots without NPK and mycorrhizae. MA+HD showed increase in number of tillers/plant by 49.5%, 33.5% and 30% over MD+HD, HD and FD respectively (*r*^2^ = 96.03%, *F* = 60.42) (Fig. 4).

### Effects of mycorrhizae inoculation on dry mass of wheat

**Figure 5:**
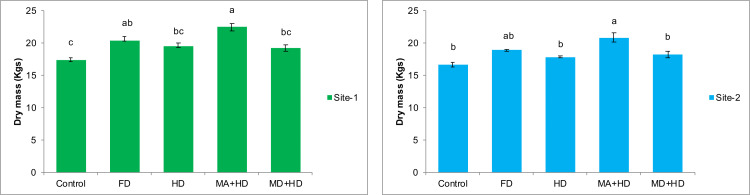
Effect of mycorrhizae on dry mass of wheat plants/plot at site-1 and site-2. Vertical bars represent standard error of means of three replications. Bars sharing same letter do not differ at *P* ≤ 0.05 as computed by ANOVA & Tukey’s test, using Minitab-19. FD. Wheat with full/recommended dose of NPK; HD. Wheat with half of recommended dose of NPK; MA+HD. Wheat with HD of NPK and mycorrhizal alive inoculum; MD+HD. Wheat with HD of NPK and mycorrhizal dead inoculum.

At site-1, MA+HD increased dry mass of wheat by 17.4%, 10.4%, and 15.8% over MD+HD, FD and HD, respectively. At site-2, MA+HD showed 14.2%, 10.5% and 16.98% increase in dry mass over MD+HD, FD and HD, respectively. In the present study, MA+HD showed 16.98% and 25.5% increase in dry biomass over, HD and control, respectively, at site-2 (Fig. 5).

### Effects of mycorrhizae inoculation on grain yield of wheat

**Figure 6:**
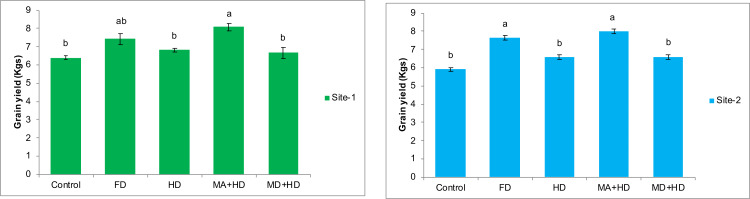
Effect of mycorrhizae on grain yield of wheat plants/plot wheat plants at site-1 and site-2. Vertical bars represent standard error of means of three replications. Bars sharing same letter do not differ at *P* ≤ 0.05 as computed by ANOVA & Tukey’s test, using Minitab-19. FD. Wheat with full/recommended dose of NPK; HD. Wheat with half of recommended dose of NPK; MA+HD. Wheat with HD of NPK and mycorrhizal alive inoculum; MD+HD. Wheat with HD of NPK and mycorrhizal dead inoculum.

The effects of various treatments on grain yield of wheat at both experimental locations are shown in Fig. 6. Treatment where field was given inoculum with mycorrhizae alive and half dose of commercial inorganic fertilizer NPK (MA+HD), maximum significant effects were recorded. There was 20.6%, 18.2%, 25.8% increase in grain yield as compared to MD+HD, HD, control, respectively. Also, at site-2, treatment, MA+HD showed significant increase in grain yield by 21.2%, 21.6% and 35.4% over MD+HD, HD, control, respectively.

### Effects of mycorrhizae inoculation on hay weight

**Figure 7:**
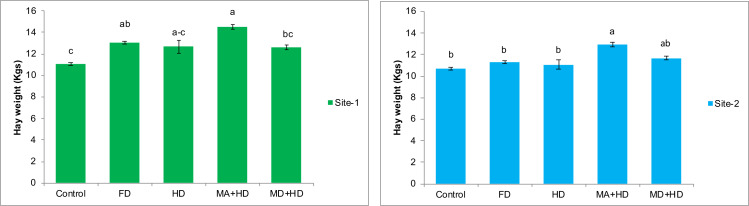
Effect of mycorrhizae on hay weight of wheat plants/plot wheat plants at site-1 and site-2. Vertical bars represent standard error of means of three replications. Bars sharing same letter do not differ at *P* ≤ 0.05 as computed by ANOVA & Tukey’s test, using Minitab-19. FD. Wheat with full/recommended dose of NPK; HD. Wheat with half of recommended dose of NPK; MA+HD. Wheat with HD of NPK and mycorrhizal alive inoculum; MD+HD. Wheat with HD of NPK and mycorrhizal dead inoculum.

In case of effects of mycorrhizal inoculation on hay weight of wheat, maximum significant increase in hay weight of wheat was recorded in treatment, MA+HD. There was 15%, 14.5%, 11.5% and 31% increase in hay weight of wheat in mycorrhizal inoculated plots (MA+HD) as compared to MD+HD, HD, FD and control, respectively. At site-2, MA+HD showed increase in hay weight by 16.7% and 14.6% over HD and FD, respectively (Fig. 7).

### Effects of mycorrhizae inoculation on 1,000 grains weight

**Figure 8:**
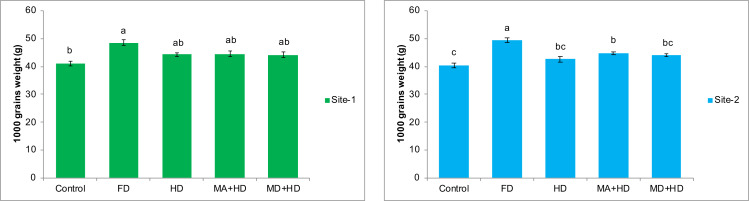
Effect of mycorrhizae on 1,000 grains weight of wheat plants at site-1 and site-2. Vertical bars represent standard error of means of three replications. Bars sharing same letter do not differ at *P* ≤ 0.05 as computed by ANOVA & Tukey’s test, using Minitab-19. FD. Wheat with full/recommended dose of NPK; HD. Wheat with half of recommended dose of NPK; MA+HD. Wheat with HD of NPK and mycorrhizal alive inoculum; MD+HD. Wheat with HD of NPK and mycorrhizal dead inoculum.

Results at both sites indicated that there was non-significant effect of all treatments on 1,000 grains weight of wheat when compared with each other except, FD, where the maximum significant increase in 1,000 grains weight was observed. Amongst all treatments, treatment where field was inoculated with mycorrhiza, and half dose of NPK, an increase of 8.6% in 1,000 grains weight of wheat was recorded when compared with plots where there was no inoculation of mycorrhizal fungi as well as no addition of NPK. The maximum increase in 1,000 grains weight was 18% as recorded in FD plots, when compared with control plots. Almost similar results were recorded at site-2 where MA+HD showed only 11.2% increase in 1,000 grains weight over control. Effect of mycorrhizal inoculation (MA+HD) did not show any significant increase in 1,000 grains weight over MD+HD at both experimental sites (Fig. 8).

### Effects of mycorrhizae inoculation on nutritional status of wheat

Protein content was significantly increased to 13.95% and 24.2% when compared with plots having half dose of synthetic fertilizers along with mycorrhiza dead (MD+HD), at site-1 and site-2, respectively. Zinc content also depicted a significant rise due to mycorrhizal inoculation. There was 24% and 21.9% increase in the zinc contents when compared with MD+HD, at site-1 and site-2, respectively. Iron, phosphorus and potassium also showed significantly increased concentrations in response to mycorrhizal inoculation. There was 21% and 15.6% rise in the concentration of iron and 21.7% and 30.9% rise in the concentration of phosphorus, while there was 14.8% and 11% enhancement in the concentration of potassium, at site-1 and site-2, respectively (Table 1).

**Table 1:** Nutritional composition of wheat grains in different treatments at site-1 and site-2.

**Treatments**	**Proteins** **(** **g/100 g)**		**Zinc** **(m** **g/100 g)**		**Iron** **(m** **g/100 g)**		**Phosphorus** **(m** **g/100 g)**		**Potassium** **(m** **g/100 g)**	
	Site-1	Site-2	Site-1	Site-2	Site-1	Site-2	Site-1	Site-2	Site-1	Site-2
Control	11 ± 0.5c	11.9 ± 0.8b	3.1 ± 0.4b	3.2 ± 0.3b	3.5 ± 0.2b	4.1 ± 0.2c	270 ± 13.2c	282 ± 7.2c	340 ± 13c	312 ± 7.2c
FD	13 ± 0.7b	13.4 ± 0.6b	3.3 ± 0.3b	3.3 ± 0.2ab	3.7 ± 0.17b	4.7 ± 0.2b	310 ± 17.1b	319 ± 5.6b	390 ± 15.6b	372 ± 12b
HD	12.8 ± 0.7b	13.0 ± 0.9b	3.2 ± 0.2b	3.1 ± 0.3b	3.8 ± 0.2b	4.5 ± 0.2bc	300 ± 9.5bc	298 ± 17.7bc	379 ± 20.1bc	360 ± 6.9b
MA+HD	14.7 ± 0.5a	15.9 ± 0.5a	4.1 ± 0.4a	4.1 ± 0.4a	4.6 ± 0.2a	5.2 ± 0.2a	365 ± 11.3a	390 ± 8.7a	435 ± 10.5a	400 ± 11.4a
MD+HD	12.9 ± 0.5b	12.8 ± 0.5b	3.3 ± 0.2b	3.2 ± 0.4b	3.8 ± 0.1b	4.7 ± 0.1b	306 ± 17.4bc	300 ± 14bc	379 ± 14bc	371 ± 12.3b

**Notes.**

Abbreviations g grams mg milli grams FD Wheat with full/recommended dose of NPK HD Wheat with half of recommended dose of NPK MA+HD Wheat with HD of NPK and mycorrhizal alive inoculum MD+HD Wheat with HD of NPK and mycorrhizal dead inoculum

Values are means of 3 replicates ± standard deviation. Standard deviation values were rounded off to first decimal. Values sharing same letter do not differ at *P* ≤ 0.05 as computed by ANOVA & Tukey’s test, using Minitab-19. Significance level was compared within each column.

### Effects of mycorrhizae inoculation on soil properties

Pre-sowing and after-harvest soil properties are given in Tables 2 and 3. After harvesting, soil organic carbon (SOC), available phosphorus (P) & potassium (K) were increased up to 53.5%, 26% and 16%, respectively, in treatments where half dose of NPK was utilized with mycorrhizal inoculum (MA+HD), at site-1, when compared with plots where half dose of NPK was utilized with mycorrhizal dead inoculum (MD+HD). However, at site-2, 64.7%, 35.8% and 23.9% increase was recorded in SOC, available phosphorus and potassium respectively, in corresponding treatments as in site-1. Besides these soil parameters, there was positive influence of adding mycorrhizal inoculum on saturation % age, soil pH and EC dSm^−1^ also, at both sites. Soil pH was decreased to 6.3 and 6.1 in mycorrhizal inoculated plots (MA+HD) when compared with MD+HD, having values of 6.9 and 7.2, at site-1 and 2, respectively. There was a significant effect of mycorrhizal addition in enhancing soil fertility. There was an increase of 30.9% and 25.3%, in saturation % age at site-1 and site-2, respectively, when MA+HD was compared with MD+HD. On the other hand, EC dSm^−1^ increased to 1.17 and 1.3 at site-1 and site-2, respectively, when MA+HD was compared with MD+HD having values of 0.81 and 0.9, respectively. The increase in saturation % age, with corresponding treatments was 25.3% at site-2. There was a significant decrease in pH and a significant increase in EC dSm^−1^ of soil at both sites due to introduction of mycorrhizal species.

**Table 2:** Pre-sowing and post-harvesting soil properties in loam soil at site-1.

**Treatments**	**Soil texture**		**Saturation % age**		**Soil pH**		**EC dSm** ^−1^		**Soil organic carbon (SOC) % age**		Available phosphorus (mg kg^−1^)		**Available potassium** (**mg kg**^−1^**)**	
	P.S.	P.H.	P.S.	P.H.	P.S.	P.H.	P.S.	P.H.	P.S.	P.H.	P.S.	P.H.	P.S.	P.H.
Control	L.	L.	39.3 ± 0.6a	36.7 ± 1.2c	7.2 ± 0.2a	6.9 ± 0.1a	0.75 ± 0.02a	0.70 ± 0.02c	0.79 ± 0.04a	0.63 ± 0.04c	4.9 ± 0.3a	4.4 ± 0.2c	120 ± 2.0a	110 ± 8.0c
FD	L.	L.	39 ± 1.7a	43 ± 1.7b	7.1 ± 0.2a	7.0 ± 0.2a	0.78 ± 0.02a	0.8 ± 0.03b	0.81 ± 0.01a	0.82 ± 0.02b	4.97 ± 0.06a	5.4 ± 0.2b	122 ± 2.1a	130 ± 3.1b
HD	L.	L.	41 ± 1.0a	41.3 ± 1.5b	7.1 ± 0.2a	7.0 ± 0.1a	0.77 ± 0.03a	0.8 ± 0.01b	0.79 ± 0.03a	0.79 ± 0.02b	4.7 ± 0.01a	5.0 ± 0.1b	123 ± 3.1a	127 ± 3.1b
MA+HD	L.	L.	41 ± 1.0a	55 ± 1.7a	7.2 ± 0.2a	6.3 ± 0.3b	0.78 ± 0.01a	1.17 ± 0.02a	0.81 ± 0.02a	1.32 ± 0.04a	4.8 ± 0.01a	6.3 ± 0.2a	118 ± 5.0a	145 ± 7.0a
MD+HD	L.	L.	40 ± 1.0a	42 ± 1.7b	7.0 ± 0.2a	6.9 ± 0.2a	0.76 ± 0.02a	0.81 ± 0.03b	0.8 ± 0.03a	0.86 ± 0.05b	4.9 ± 0.2a	5.0 ± 0.2b	119 ± 3.6a	125 ± 3.0b

**Notes.**

Abbreviations P.S. Pre-sowing P.H. Post harvesting L loam NPK Nitrogen, phosphorus, and potassium EC dSm^−1^ Electrical conductivity units are deciSiemens per metre (dS/m) FD Wheat with full/recommended dose of NPK HD Wheat with half of recommended dose of NPK MA+HD Wheat with HD of NPK and mycorrhizal alive inoculum MD+HD Wheat with HD of NPK and mycorrhizal dead inoculum

Values are means of 3 replicates ± standard deviation. Standard deviation values were rounded off to 1^st^ and 2^nd^ decimal, where considered appropriate. Values sharing same letter do not differ at *P* ≤ 0.05 as computed by ANOVA & Tukey’s test, using Minitab-19. Significance level was compared within each column.

**Table 3:** Pre-sowing and post-harvesting soil properties in loam soil at site-2.

**Treatments**	**Soil texture**		**Saturation % age**		**Soil pH**		**EC dSm** ^−1^		**Soil organic carbon (SOC) % age**		**Available phosphorus (mg kg**^−1^)		**Available potassium** (mg kg^−1^)	
	P.S.	P.H.	P.S.	P.H.	P.S.	P.H.	P.S.	P.H.	P.S.	P.H.	P.S.	P.H.	P.S.	P.H.
Control	L.	L.	44.8 ± 0.6a	41 ± 1.0c	7.3 ± 0.3a	7.2 ± 0.2a	078 ± 0.03a	0.72 ± 0.03d	0.85 ± 0.03a	0.59 ± 0.01c	4.8 ± 0.2a	4.0 ± 0.17c	133 ± 4.3a	112 ± 4.4c
FD	L.	L.	45 ± 1.0a	48 ± 1.5b	7.4 ± 0.2a	7.3 ± 0.3a	0.8 ± 0.03a	0.82 ± 0.02bc	0.86 ± 0.04a	0.89 ± 0.05b	4.83 ± 0.2a	5.2 ± 0.3b	136 ± 3.6a	143 ± 4.4b
HD	L.	L.	43.7 ± 1.5a	42 ± 1.0c	7.3 ± 0.1a	7.1 ± 0.02a	0.8 ± 0.02a	0.81 ± 0.02c	0.86 ± 0.03a	0.85 ± 0.05b	5.1 ± 0.3a	5.3 ± 0.40b	131 ± 5.3a	133 ± 3.2b
MA+HD	L.	L.	43 ± 1.0a	61 ± 2.6a	7.2 ± 0.3a	6.1 ± 0.3b	0.8 ± 0.03a	1.3 ± 0.02a	0.85 ± 0.03a	1.40 ± 0.07a	5.0 ± 0.2a	7.2 ± 0.3a	134 ± 4.4a	166 ± 4.6a
MD+HD	L.	L.	45.3 ± 2.5a	48.7 ± 0.9c	7.3 ± 0.3a	7.2 ± 0.2a	0.79 ± 0.02a	0.9 ± 0.05b	0..85 ± 0.03a	0.85 ± 0.03b	5.1 ± 0.17a	5.3 ± 0.2b	129.3 ± 3.1a	134 ± 2.3b

**Notes.**

Abbreviations P.S. Pre-sowing P.H. Post harvesting L. loam NPK Nitrogen, phosphorus, and potassium EC dSm^−1^ Electrical conductivity units are deciSiemens per metre (dS/m) FD Wheat with full/recommended dose of NPK HD Wheat with half of recommended dose of NPK MA+HD Wheat with HD of NPK and mycorrhizal alive inoculum MD+HD Wheat with HD of NPK and mycorrhizal dead inoculum

Values are means of 3 replicates ± standard deviation. Standard deviation values were rounded off to 1^st^ and 2^nd^ decimal, where considered appropriate. Values sharing same letter do not differ at *P* ≤ 0.05 as computed by ANOVA & Tukey’s test, using Minitab-19. Significance level was compared within each column.

## Discussion

Arbuscular mycorrhizal fungi (AMF) are considered as an optimal eco-friendly and biological technique to increase crop yield to address food security (Jerbi et al., 2022; Khan, Shah & Tian, 2022). Enhancing grain yield of cereals like wheat is vital in agriculture (Zhang et al., 2019a; Zhang et al., 2019b). Use of AMF can increase the concentrations of macro- and micro nutrients, thereby enhancing photosynthates leading to higher biomass production (Chen et al., 2017; Mitra et al., 2019). In the present study, significant enhancement effects were recorded in the growth of wheat at both experimental sites, by the application of inorganic fertilizers as well as mycorrhizal inoculation. The application of fertilizer and AMF, both increased the height and tillers/plant in wheat but in contrast to increase in height of wheat, introduction of AMF conspicuously increased tillers/plant in wheat. As the mycorrhizal inoculation triggered the tiller formation in wheat, but 1,000 grain weight was not affected by AMF inoculation which provided conclusive evidence of tillering enhancement in wheat under the influence of mycorrhizal inoculation in wheat. Mycorrhizal inoculation effect on tiller formation in wheat, but no effect on 1,000 grain weight can be explained as AMF colonization in wheat can affect gene expression/transcription profile of the plant growth (Moradi Tarnabi et al., 2020), but it still remained to be investigated. Khan & Zaidi (2007) also reported that when the inoculation of *Glomus fasciculatum* was made with wheat seeds, a significant increase of 2.6 folds in dry matter was observed. Al-Karaki, McMichael & Zak (2004), reported that there was variable contribution of increase in number of heads in wheat to overall wheat biomass as well as grain yield as compared to increase in grain yield of wheat, when inoculated with *Glomus etunicatum* and *Glomus mosseae.* Moreover, there was also variable response when the effectiveness of the two species of mycorrhizae was considered. *G. etunicatum* inoculated wheat plants generally had higher biomass and grain yield than those wheat plants inoculated with *G. mosseae*. Bangash et al. (2013), also indicated that inoculation with biofertilizers having mycorrhizal fungi and plant growth promoting rhizobacteria (PGPR) strains, *Serratia* and *Aerococcus,* improved the growth of wheat seedlings. There was significant enhancement in the root and shoot length, root and shoot dry mass by 54%, 80%, 54%, and 95%, respectively, over un-inoculated control.

Moreover, nutritional status of wheat grains was significantly improved under the influence of mycorrhiza. The application of N fertilizer can boost both yield and protein % age (Fowler et al., 1990). The addition of P is well known to significantly increase plant growth and grain yield in wheat (Li et al., 2005). AMF can also modify grain nutrient concentrations in wheat (Watts-Williams & Gilbert, 2021; Yadav, Chakraborty & Ramakrishna, 2022). In another investigation, there was 28, 50, and 30% increase in tiller dry weight, grain yield/spike and protein % age of grains of wheat by the inoculation of mycorrhiza in wheat (Allah et al., 2015). AMF have shown increased K and P concentrations, resulting in increased crop growth (Balliu, Sallaku & Rewald, 2015). AMF establish symbiotic relationship with roots to obtain nutrients from the host plant and in return provide mineral nutrients *e.g.*, P and K. AMF produce arbuscules, which perform exchange of minerals and the compounds of carbon and phosphorus in plants (Li, Zeng & Liao, 2016; Prasad et al., 2017). Grain yield (27%), protein (4%), Fe (8%), and Zn (36%) were recorded in chickpea inoculated with mycorrhiza (Pellegrino & Bedini, 2014). The enhancement effects on yield as well as nutritional attributes can be attributed to various physiological processes carried out by mycorrhizae *e.g.*, *Glomus mosseae* increased chlorophyll contents, enzymes of N and P metabolism, and NPK in *Triticum aestivum* (Rani, 2016). *Rhizophagus intraradices* inoculation resulted in higher grain yield, and contents of Cu, Fe, Mn, Zn and gliadins (protein) in grains of *Triticum durum* (Goicoechea et al., 2016). *Claroideoglomus etunicatum* increased plant growth, free *α*-amino acids, and Na^+^ and K^+^ uptake in *Aeluropus littoralis* (Hajiboland, Dashtebani & Aliasgharzad, 2015). Inoculation by *G. mosseae* in wheat increased uptake of P, K and Zn by 35%, 31.8% and 18%, respectively (Daei et al., 2009). AMF inoculation increased grain yield in wheat genotypes by 24% and this increased grain yield resulted from increased number of spikes per unit area. There was nonsignificant effect of AMF on wheat grain weight. There was a 16%, 44% and 30% increase in protein content, P and Fe in wheat grains. However, the increase in nutritional contents in wheat was variety dependent (De Santis et al., 2022).

AMF are ubiquitous symbionts which increase plant nitrogen acquisition (Hestrin et al., 2019). In a previous study, increase in the concentration of proteins in wheat was also recorded due to inoculation of AMF. Moreover, significant improvements were also recorded in relative water content % age, membrane stability index % age, increased net CO_2_ assimilation rate, stomatal conductance and concentration of sodium, nitrogen, magnesium and potassium. This increase can be attributed to increased chlorophyll contents, leading to enhanced photosynthesis, enhanced metabolism of carbon and nitrogen, leading to significantly higher grain yield in wheat by 75% and 47.6%, when compared with non mycorrhizal wheat varieties Sids 1 and Giza 168, respectively (Talaat & Shawky, 2014). The positive role of mycorrhizae on nutrient uptake in wheat has been well documented (Ganugi et al., 2019). A meta-analysis conducted on 38 field trials highlighted the beneficial effects of mycorrhizal inoculation on wheat dry weight and uptake of P, N, and Zn (Pellegrino et al., 2015). In another study, mycorrhizal fungi significantly increased N, P and K contents in wheat shoot by 58.2%, 48.98% and 30.96%, respectively (Elgharably & Nafady, 2021). Another study suggested that the mycorrhizal inoculated wheat had higher shoot P & Fe concentrations than non-inoculated wheat plants (Al-Karaki, McMichael & Zak, 2004).

There is lack of host- and niche-specificity in AMF, indicating that AMF are feasible for use in a wide range of ecological conditions (Huey et al., 2020). In the present investigation, the effects of AMF inoculation on soil properties were also encouraging at both experimental sites. AMF are key factors of the soil/plant system, influencing soil fertility and plant nutrition, and contribute to soil aggregation and soil structure stability (Bedini et al., 2009). Soil organic matter (SOM) acts as nutrition for plants. Furthermore, much of the accumulated C originate largely from root-associated fungal hyphae. Mycorrhizal colonization alters C allocation patterns within the host plant and changes the quantity and quality of C entering SOM pools (Frey, 2019). AMF hyphae increase mineralization on native SOM (Paterson et al., 2016). AM fungi can almost double soil carbon content percentage in just one year, while no increase in soil organic carbon (SOC) was observed with tall fescue grass without AM fungal inoculation (Amaranthus et al., 2022). AMF also have a +ive effect on the soil by producing organic acids and glomalin, which protect from soil erosion, improve carbon sequestration, and soil macro-aggregation. AMF also recruits bacteria that produce alkaline phosphatase, associated with phosphorus availability (Fall et al., 2022). In an investigation, after 150 days of AMF inoculation, the levels of SOC, in AMF treatments were significantly enhanced by 52–61%, in comparison with control. These data reveal that AMF infection increased organic matter and glomalin which can be linked with the increase of SOC in soil (Wang et al., 2016). In another study, AMF inoculation increased the total organic matter in soil by 24.97%, under drought stress, while under well-watered conditions, this value was 13%. On the other hand, phosphorous increased up to 620.9% and 166.4% under drought stress and well-watered conditions, respectively, in soil of quinoa plants, under field conditions (Benaffari et al., 2022). *Rhizophagus irregularis* and *Glomus versiforme* increased easily extractable glomalin reactive soil protein (EE-GRSP) contents, while *G*. *versiforme* had a greater effect of about 400%, in comparison with control, in EE-GRSP accumulation in soil. Moreover, EE-GRSP/SOC %, in *G*. *versiforme* treatment was about 300% (Zhang et al., 2019a; Zhang et al., 2019b). The mycorrhizal external mycelia are the dominant pathway (62%) through which carbon enters the SOM pool of soil, and this contribution exceeds the input through leaf litter and fine root turnover (Godbold et al., 2006). Due to rapid turnover, dead microbial biomass can make a disproportionately large contribution to total SOM relative to the amount of standing microbial biomass (Grandy & Neff, 2008). Nitrogen availability is regarded as a limiting factor in plant growth. In another investigation, mycorrhizae + vermicompost significantly reduced soil pH by 5% and 6%, increased organic matter by 25% and 112%, total N by 41%, and P up to 200% (Hussain et al., 2018).

In the text, effect of mycorrhizal addition (MA+HD) was compared to plots where mycorrhizal dead inoculum (MD+HD), considering the fact that better picture of the experiment can be seen when these two treatments are compared with each other. On the other hand, when we compare the effect of mycorrhizal addition with other treatments *e.g.*, control, FD, or HD, the effects of mycorrhizal addition can be seen much enhanced. In previous investigations, this way to calculate the effect of mycorrhizal inoculation in wheat crop has not been addressed, so, our study gives a better picture of effects of different treatments on the growth parameters of wheat and effects on soil fertility indicators.

The results of the present research depict that mycorrhizal inoculations are the best for organic farming, as these mycorrhizae not only improve the quantity of yield but quality also, because mycorrhizal inoculation reduces the input of commercial inorganic fertilizer. The role of mycorrhizae in maintaining and increasing soil fertility has also been described by other workers (Reeve et al., 2016). The slight differences observed in wheat growth parameters as well as soil fertility factors can be attributed to variations in the working of AM fungi. Different wheat varieties show variable response to inoculation with AM fungi isolated from organic and conventional agricultural fields. The use of AMF from organic fields resulted in slightly taller plants. Pikker wheat cultivar exhibited relatively higher yield and stronger growth when the organic AMF was used. Arabella wheat cultivar showed relatively less yield and poor growth when the organic AMF was utilized (García de León et al., 2020). Spiked levels of available P and K are very useful for the growth of wheat plants in sustainable agriculture because the introduction of mycorrhizae not only improves soil properties in current inoculated crop but enhanced SOC, available P and K are beneficial for the next crop. In the present study, native mycorrhizal species were investigated for their efficacy to boost wheat yield and the use of native mycorrhizal species has been considered helpful for proper functioning of the ecosystem (Middleton & Bever, 2012; Koziol et al., 2018). Native plants depend on the native soil microbial communities including AM fungi and any disturbance in native microbial composition may help the non-native plants to invade that area (Wilson, Hickman & Williamson, 2012; Wilson & Hartnett, 1998; Vogelsang & Bever, 2009). Loss of AMF symbiosis due to disturbance by non-native plants may reduce AM diversity and fungal propagules available to native species, with resultant loss of native plant species that are dependent on locally adapted AMF (Wagg et al., 2011). In addition, AMF improve soil carbon storage and aggregate stability therefore, loss of AMF hyphae declines soil carbon storage and aggregate stability (Wilson et al., 2009). Application of native AMF improves plant tolerance to abiotic factors and promote activities of antioxidant enzymes, thereby increasing plant development (Outamamat et al., 2021).

## Conclusions

The predominant mycorrhizal species identified from the experimental areas belonged to genus, *Claroideoglomus*. Bio-inoculation of consortia of different mycorrhizal species showed a significant increase in growth parameters of wheat, especially, number of tillers/plant (up to 49.5%) and grain yield (up to 21.2%). However, there was non-significant effect of mycorrhizal inoculation on 1,000 grains weight, which provided evidence that mycorrhizal species enhanced tillering in wheat at both sites, thereby showing an increased wheat yield. Proteins, zinc, iron, phosphorus and potassium concentrations in wheat grains were increased to 24.2%, 24.2%, 24%, 21%, 30.9% and 14.8%, respectively. Moreover, notable effects were observed on soil fertility such as soil organic carbon, phosphorus and potassium were increased up to 64.7%, 35.8% and 23.9%, respectively. The present study recommends AMF as future alternative to synthetic fertilizers. Moreover, the underlined mechanisms involved in increased tillering in wheat in response to mycorrhizae need to be investigated.

##  Supplemental Information

10.7717/peerj.15686/supp-1 Supplemental Information 1 Raw data

## Additional Information and Declarations

### Field Study Permissions

The following information was supplied relating to field study approvals (i.e., approving body and any reference numbers): Permission was granted by farmers (Muhammad Afzal, Muhammad Arif) to access field sites.

### Data Availability

The following information was supplied regarding data availability: The raw data are available in the Supplemental Files.
